# Missed Pharyngeal Foreign Body in an Infant that Persisted for 50 days:A Rare Case

**Published:** 2012

**Authors:** Mohammad Saeed Ahmadi, Mohammad Ahmadi

**Affiliations:** 1 *Department of otorhinolaryngology, Hamadan University of Medical Sciences, Hamadan, Iran*; 2 *Otorhinolaryngology assistance *

**Fig 1:**
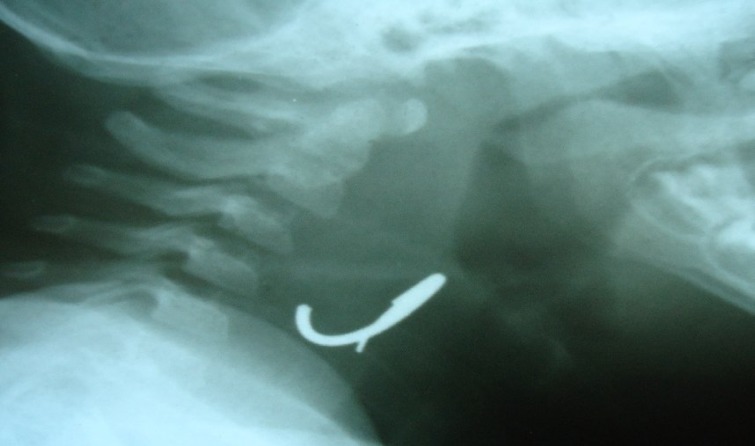
X-ray of the Pharyngeal Region Showing a Metallic Density

In this case report we present a rare case of a longstanding undetected foreign body in the pharynx. A 10-month-old girl presented with a history of vomiting and drooling. This complaint had first been noticed 50 days previously. It was noted that she had been diagnosed with upper respiratory or gastrointestinal tract infections several times during the previous 50 days and that symptomatic treatment had shown no benefits. The results of a physical examination were unremarkable and weight loss was only slight. Radiography of the neck region showed a metallic ring stuck to the pharyngeal wall (Fig. 1). 

The parents then reported that at the age of 8 months a golden ring went missing while the child was playing with it. 

**Fig 2:**
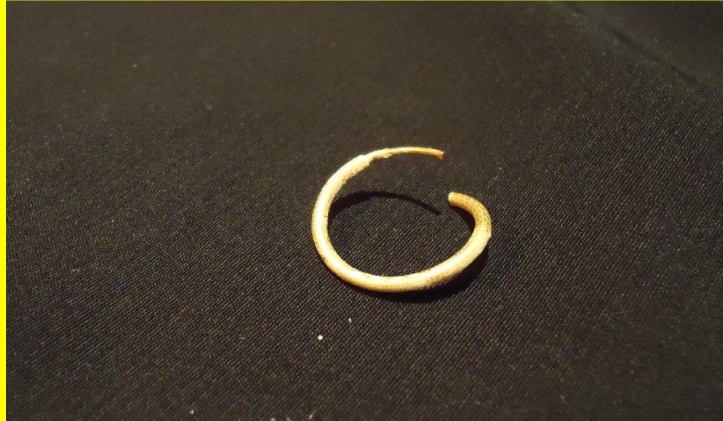
The foreign body

During an endoscopic examination under general anesthesia, it was noted that above the cricopharyngeus there was a granulated tumefaction surrounding a golden ring, which was removed (Fig. 2). No complications were observed.

Ingestion of foreign bodies is a common complaint in pediatric practice. The items swallowed are usually toy parts, needles, buttons and so on. They usually pass harmlessly through the gastrointestinal tract but a few become impacted in the pharyngeal wall. The diagnosis of a pharyngeal foreign body may pose a problem, as in the patient described here. The symptoms are usually dysphagia, pain, drooling, upper respiratory tract infection, or refusal to eat. However, only vomiting and drooling were observed in our patient. Undiagnosed pharyngeal foreign bodies can result in retropharyngeal cellulitis or abscess and mediastinitis. 

A thorough patient history can provide a clue to the diagnosis, but if the history is not reliable enough, as in our patient, simple radiographic evaluation of the upper airway may provide information to the diagnostician. In our case, radiography had not performed during the initial management of the case. For that reason, there was a delay in the diagnosis of the foreign body. In conclusion, it is important to be aware of the possibility of a pharyngeal foreign body in children, particularly when the history is unreliable or if the clinical symptoms are atypical. 

